# The Efficacy of Emamectin Benzoate against Infestations of *Lepeophtheirus salmonis* on Farmed Atlantic Salmon (*Salmo salar* L) in Scotland, 2002–2006

**DOI:** 10.1371/journal.pone.0001549

**Published:** 2008-02-06

**Authors:** Fiona Lees, Mark Baillie, George Gettinby, Crawford W. Revie

**Affiliations:** 1 Department of Statistics and Modelling Science, University of Strathclyde, Glasgow, United Kingdom; 2 Department of Computer and Information Sciences, University of Strathclyde, Glasgow, United Kingdom; Monash University, Australia

## Abstract

**Background:**

Infestations of the parasitic copepod *Lepeophtheirus salmonis*, commonly referred to as sea lice, represent a major challenge to commercial salmon aquaculture. Dependence on a limited number of theraputants to control such infestations has led to concerns of reduced sensitivity in some sea lice populations. This study investigates trends in the efficacy of the in-feed treatment emamectin benzoate in Scotland, the active ingredient most widely used across all salmon producing regions.

**Methodology/Principal Findings:**

Study data were drawn from over 50 commercial Atlantic salmon farms on the west coast of Scotland between 2002 and 2006. An epi-informatics approach was adopted whereby available farm records, descriptive epidemiological summaries and statistical linear modelling methods were used to identify factors that significantly affect sea lice abundance following treatment with emamectin benzoate (SLICE®, Schering Plough Animal Health). The results show that although sea lice infestations are reduced following the application of emamectin benzoate, not all treatments are effective. Specifically there is evidence of variation across geographical regions and a reduction in efficacy over time.

**Conclusions/Significance:**

Reduced sensitivity and potential resistance to currently available medicines are constant threats to maintaining control of sea lice populations on Atlantic salmon farms. There is a need for on-going monitoring of emamectin benzoate treatment efficacy together with reasons for any apparent reduction in performance. In addition, strategic rotation of medicines should be encouraged and empirical evidence for the benefit of such strategies more fully evaluated.

## Introduction

Commercial farming of Atlantic salmon (*Salmo salar* L) has developed rapidly since the 1970's, with global production exceeding one million tonnes per annum since 2002 [1]. Atlantic salmon farming is currently dominated by the aquaculture industries of Norway and Chile, however Scotland and Canada are also major producers.

As intensive marine aquaculture developed, the threat posed to fish health and production by infestations of parasitic copepods emerged as one of the greatest challenges facing the industry [2]. Not only can these aquatic parasites inhibit growth and cause extensive damage, extreme infestation can lead to host mortality [3]. It has also been suggested that caligid copepods, commonly referred to as sea lice, originating from salmon farms may pose a risk to wild salmonid populations [4]–[8].

In Scotland two species of sea lice parasitise farmed salmonids: *Lepeophtheirus salmonis* (Krøyer 1837) and *Caligus elongatus* (Nordmann 1832). Of the two species, *L. salmonis* is the larger and more abundant [9]. Whereas *C. elongatus* is known to parasitise more than 80 species of fish, the major species of interest *L. salmonis* is principally confined to salmonids [10].

In response to the challenges presented by sea lice infestation, salmon producers on the west coast of Scotland have developed integrated health management programmes based on previous research into the epidemiology of sea lice [11]–[14] and farm management practices [15]–[18]. Some of these management strategies have proven to be successful and, together with the availability of more effective ectoparasitic medicines, have helped to reduce the abundance of *L. salmonis* and *C. elongatus* on Scottish farms over the past decade [19]. Nevertheless, sea lice remain a persistent problem and the cost of controlling these parasites is substantial [18].

The availability and use of medicines to control sea lice burdens in Scotland has changed considerably in the last decade and since 2005 only two therapeutants have been in common use; the topical treatment cypermethrin (Excis®, Novartis Animal Health) and the in-feed treatment emamectin benzoate (SLICE®, Schering Plough Animal Health). Both ectoparasiticides are widely used and, since obtaining UK Market Authorisation in 2000, the use of emamectin benzoate (SLICE®) has grown dramatically [19]. While both medicines are effective against all parasitic stages of sea lice, the major advantage of emamectin benzoate is that it can offer sustained periods of louse clearance [20]–[22]. Furthermore, as an in-feed therapeutant, it can be safely and effectively administered during adverse weather conditions and whole sites/loch systems can be medicated in a coordinated manner. Cypermethrin bath treatments are more labour intensive and can be stressful to the fish; these interventions are applied more frequently toward the end of the production cycle when salmon are larger and in-feed treatments are consequently more costly.

In Canada, emamectin benzoate (SLICE®) is available under Emergency Drug Release and another in-feed medicine, teflubenzuron (Calicide®, Skretting), has an INAD (Investigational New Animal Drug) approval. However, salmon producers in British Columbia only have access to emamectin benzoate [23]. Until recently, various brands of emamectin benzoate were the only sea lice medicines available in Chile, but the topical treatment deltamethrin (Alpha Max®, Pharmaq) was licensed for use in late 2007. It should be noted that Atlantic farmed salmon in this region are parasitised by *Caligus rogercresseyi* (Boxshall and Bravo 2000) rather than *L. salmonis* and *C. elongatus*. Dependence on such a limited range of ectoparasiticides has raised concerns that resistance in lice will become an increasing problem for salmon farmers unless new medicines become available and/or existing therapeutants are appropriately managed [24], [25]. While there have been anecdotal reports of reduced sensitivity and potential resistance of sea lice to emamectin benzoate, particularly in Chile, few published studies have examined the efficacy of emamectin benzoate on infestations of *L. salmonis* in farmed Atlantic salmon since regular treatment began in Scotland in 2002 [26], [27].

As described in a recent report by Gustafson *et al* [28], efficacy studies usually rely on the availability of an untreated control group with which to compare medicated subjects. However, in a commercial farm setting where untreated salmon could suffer extensive damage from sea lice infestation this option can rarely be justified. In this situation an alternative strategy is to monitor on-going efficacy by comparing post-treatment lice abundance with levels recorded prior to treatment intervention [23], [26], [28].

Using an epi-informatics approach, this study aims to examine the efficacy of emamectin benzoate against infestations of the mobile stages of *L. salmonis* in a commercial setting over a five-year period. Through examining sea lice abundance and treatment records drawn from over 50 salmon farms located along the west coast of Scotland between 2002 and 2006, this study identifies factors associated with the efficacy of emamectin benzoate treatment interventions.

## Materials and Methods

### Data set

Sea lice abundance and treatment data, in addition to site stocking records, were drawn from 56 commercial Atlantic salmon (*Salmo salar* L) farms located along the west coast of Scotland between 2002 and 2006. As in earlier analyses, [19], [29] sites were divided above and below the 57°N line of latitude and referred to as “North” and “South” regions respectively. Those sites noted as “Western Isles” included farms on the east coast of South Uist and on both the east and west coasts of Harris and Lewis. All farms had implemented an integrated health management programme that included the routine monitoring of sea lice abundance throughout the production cycle. All sites were owned and managed by Marine Harvest (Scotland).

Sites were stocked with a single year class of fish and for the most part operated an 18 to 24 month production cycle. Typically farms were stocked between January and June of the first production year and harvested between August and December of the second. At some sites, fish were introduced in October and others were not harvested until spring of the third year. Occasionally stocks were split at the end of the first production year. The number of stocked sites varied from year to year, but all farms were fallowed for a minimum of six weeks between production cycles.

Treatment records provided the start and end date of each sea lice treatment episode in addition to the type of medicine(s) applied and the quantity administered. The number of pens treated was recorded which, when compared with stocking data, allowed each episode to be classified as either a full or partial site treatment. Stocking records also made it possible to categorise treatments as having taken place in the first or second year of production. Episodes were further classified by the season in which they occurred with months grouped together according to mean sea water temperatures. In this analysis, spring is defined as February to April; summer as May to July; autumn as August to October; and winter as November to January. Water temperatures were lowest in the spring months and highest in the autumn.

Where possible the industrial partner (Marine Harvest) performed routine lice counts weekly, however because monitoring can at times be logistically difficult, e.g. due to adverse weather conditions, farms sometimes deviated from this protocol and observations at some locations became more sporadic. Depending on the size of the farm, 10 to 30 fish from between two and six pens were randomly monitored for lice at each sample point [30], [31]. Fish were removed by dip net and anaesthetised before being visually inspected for lice. Lice counts were classified according to species with *L. salmonis* further differentiated by one of five gender/life-stages: chalimus, pre-adult, adult male, non-gravid female and gravid female. In the analyses presented here the pre-adult, adult male, non-gravid female and gravid female stages of *L. salmonis* have been aggregated and are reported as *L. salmonis* mobiles. The mobile stages, so called due to their ability to move around and between fish, are the stages against which treatments are typically targeted as they tend to cause the greatest damage to the host.

### Treatment episode selection

In the period 2002 to 2006, 561 sea lice treatment episodes were available for analysis, 258 of which included the use of the in-feed medicine emamectin benzoate (SLICE®). All but one of the sites studied had at least one treatment episode involving this medicine.

To ensure consistency only site-wide treatment episodes, where fish in all pens began treatment on the same day, were selected for inclusion within this analysis. Mixed treatment episodes, where some pens were medicated with emamectin benzoate and other pens with a different theraputant, were not included. Treatments that occurred on sites that did not follow the typical two-year production cycle, i.e. those that were stocked between July and August, were also excluded from this analysis because such treatments could not readily be classified as first or second year interventions. All emamectin benzoate treatments were administered as medicated feed at 50 µg kg^−1^ fish for 7 consecutive days.

In contrast to some other regions [28], strategic sea lice treatments are encouraged in Scotland; often in the early spring of the second year of production [32]. On some occasions, such interventions are made even when lice levels are low and mean louse abundance is below treatment-trigger guidelines (see Table 1). The aim of strategic treatment is to disrupt the life-cycle of *L. salmonis* and prevent levels rising in the latter part of the production cycle. Such treatments are typically co-ordinated across whole loch systems, or other pre-agreed geographical areas, to minimise the risk for cross infestation amongst sites.

**Table 1:** Definition of terms used within emamectin benzoate efficacy analysis.

Term	Definition
Emamectin benzoate treatment:	Any site-wide treatment episode where emamectin benzoate was the only sea lice medicine administered and all pens commenced treatment on the same day.
Effective treatment:	Any emamectin benzoate treatment where the mean abundance of mobile *L. salmonis* fell below 40% of pre-treatment levels in the 12 weeks following treatment. Note: All treatments that were applied before guideline treatment-trigger lice levels had been reached were classified as effective.
Loch-wide treatment:	All stocked farms (owned by the industrial partner) within a loch system were treated for sea lice within two weeks of each other. The sea lice medicine used at the other sites in the loch system was not necessarily emamectin benzoate.
Effective loch-wide treatment:	Any effective treatment that was administered as part of a loch-wide intervention. The sea lice medicine used at the other sites in the loch system was not necessarily emamectin benzoate. The treatment episodes at the other sites in the loch system were not necessarily effective.
Treatment-trigger guidelines:	Mean abundance of *L. salmonis* adult females has reached 0.5 (February to June), or 1.0 (July to January).

### Efficacy calculations

Treatment efficacy was investigated by comparing post-treatment mobile *L. salmonis* abundance with levels recorded prior to treatment intervention. To establish a meaningful pre-treatment figure with which to compare post-treatment abundance, at least one lice count had to be available for a site in the 16 day period prior to treatment. If lice abundance was monitored more than once during this time, then levels recorded on the date closest to the point of treatment intervention were used as the baseline. To permit analysis at various time points after treatment, only emamectin benzoate episodes where lice levels were monitored in at least three of the 12 weeks following treatment, or before another treatment was applied, were included.

Where treatment efficacy is summarised by year, post-treatment lice abundance was examined in two ways: as mean lice per fish; and as a percentage of pre-treatment abundance (mean post-treatment abundance/mean pre-treatment abundance * 100). When using the latter approach it was important to ensure that the percentage change was based on matched pre and post-treatment lice counts. This allowed the percentage change to be estimated for each seven day period in the 83 days following treatment, even though lice levels were not always monitored at all treated sites every week or for the full 12 weeks following treatment. It should be noted that not all treatment episodes included in this analysis were deemed to have been “effective” (Table 1).

### Data management and statistical analysis

Data were stored and managed using a set of structured tables in Microsoft Access 2003. This application was also used to calculate the mean sea lice abundance values and post-treatment lice abundance as a percentage of pre-treatment levels. All figures were constructed using Microsoft Excel 2003 while statistical analyses were performed within Minitab 14.1.

A statistical model to determine the factors affecting post-treatment lice abundance was investigated using a General Linear Model (GLM) procedure. Interactions between all available factors were also examined. As treatment lasted for 7 days (day 0 to 6), post-treatment abundance was only analysed for the 7 to 83 day period following treatment initiation. To improve normality and equalise variances, data were logarithmically transformed (ln(x+1)) prior to GLM analysis. To aid interpretation of the final models, least squares means and their 95% confidence intervals were subsequently untransformed.

## Results

The sea lice treatment screening process resulted in a data set containing 185 emamectin benzoate treatment episodes, 77 of which did not have the necessary pre/post treatment sea lice data required for further efficacy analysis. Of the remaining 108 treatments, 22 were followed-up with another sea lice treatment (either cypermethrin or emamectin benzoate) within 12 weeks.

A summary of the treatment episodes in each of the data sets is presented in Table 2. The number of treatment episodes, and sites on which they were administered, varied over the five-year period studied. However, the majority (84%) of sites studied were represented in the final data set used for efficacy analysis.

**Table 2:** Numbers of sites and episodes of emamectin benzoate treatment in years 2002 to 2006.

	All treatments		Treatments with necessary pre/post treatment sea lice data	
Year	No. of sites	No. of episodes	No. of sites	No. of episodes
				
2002	22	26	10	10
2003	24	31	18	21
2004	35	56	24	31
2005	31	47	25	30
2006	17	25	13	16
**All**	**54**	**185**	**47**	**108**

Figures are presented for all site-wide emamectin benzoate treatment episodes and for episodes with the necessary pre/post treatment sea lice data required for further analysis.

**Figure 1:**
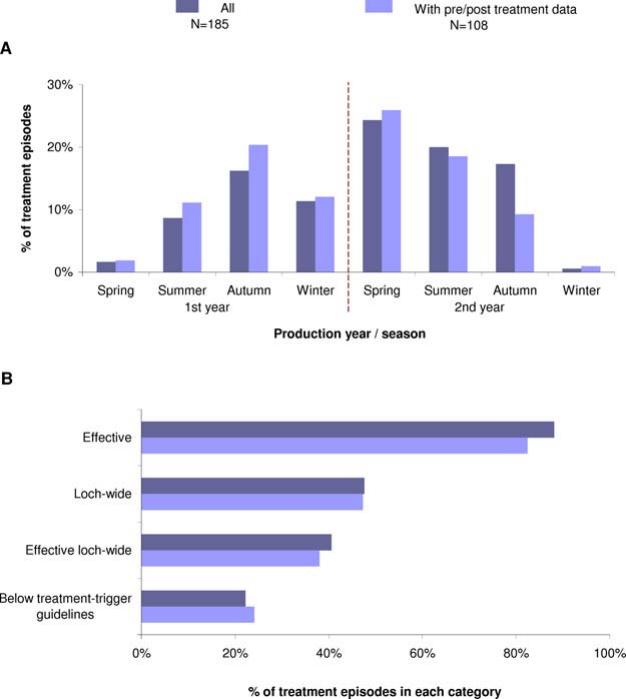
Profile of emamectin benzoate treatment episodes in years 2002 to 2006. Proportions are presented for all site-wide emamectin benzoate treatment episodes and for episodes with the necessary pre/post treatment sea lice data required for further analysis. (A) The proportion of treatment episodes occurring in each stage of production/season in the two year production cycle. Seasons are defined as spring [Feb–Apr], summer [May–Jul], autumn [Aug–Oct] and winter [Nov–Jan]. (B) The proportion of effective, loch-wide and effective loch-wide treatment episodes and the proportion of treatments applied when lice levels were below treatment-trigger guidelines.

As shown in Figure 1, the profiles of the full and the reduced data sets were broadly similar. In the first year of production the majority of emamectin benzoate treatments were administered in the autumn (Aug–Oct) whereas in the second year most occurred in the spring (Feb–Apr). Few episodes occurred in spring of the first year or winter (Nov–Jan) of the second. The main distinction between the full and the reduced data sets was the proportion of episodes administered in the autumn of the second production year. Several of the emamectin benzoate treatments applied in this period had to be discounted from the efficacy analysis because they were followed up with at least one cypermethrin treatment within a matter of weeks. The other difference of note was the lower proportion of effective treatments present within the reduced data set. However, of the 77 treatments that were excluded from further analysis, it remains unclear how effective they were due to limited lice count data prior to and/or following treatment. Similarly, without a valid pre-treatment lice count, it could not be ascertained how many adult female lice were present and therefore whether guideline treatment-trigger levels had been reached. Thus the proportions shown for the full data set should be regarded as estimates, as the true incidence of effective episodes may have been lower and the frequency with which treatments were applied when lice levels were below trigger guidelines may have been higher.

### Trends in treatment efficacy

**Figure 2:**
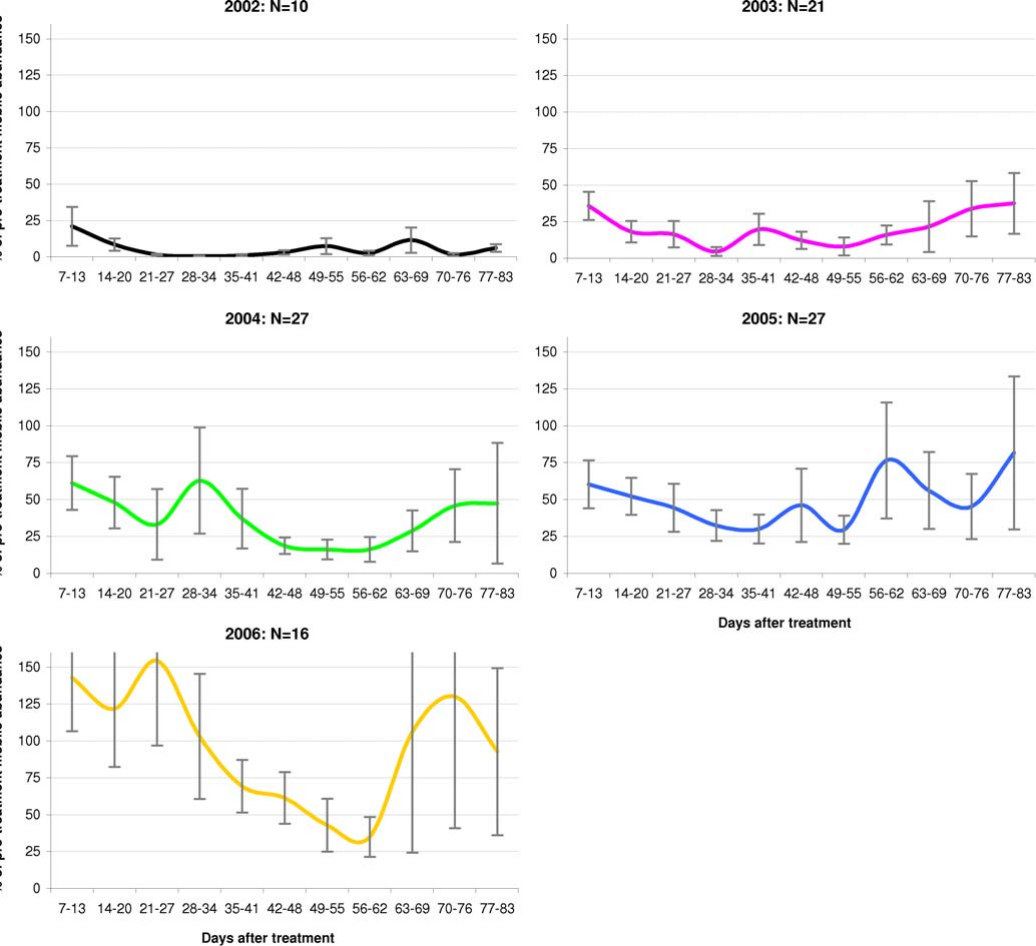
Efficacy of emamectin benzoate treatments in controlling infestations of mobile *Lepeophtheirus salmonis,* between 2002 and 2006. Post-treatment mobile *L. salmonis* abundance as a percentage of pre-treatment abundance (±SE), 7–83 days after commencement of treatment. Plots based on data from 101 treatment episodes at 47 Atlantic salmon farms in Scotland in the period 2002 to 2006.

The mean annual efficacy of emamectin benzoate treatments in controlling infestations of mobile L. salmonis, in the 83 day period following treatment initiation, is shown in Figure 2. It should be noted that efficacy percentages could not be calculated for episodes with a pre-treatment mobile abundance of zero. This resulted in four treatment episodes from 2004 and three from 2003 being excluded from this summary plot.

With the exception of 2006, mean louse abundance in all years fell to less than 45% of pre-treatment levels within 27 days of treatment intervention. In 2002, abundance fell below 1% of pre-treatment levels within 34 days and remained lower than 12% throughout the rest of the 83 day period. Treatments applied in 2003 also appeared to be highly efficacious, with abundance falling below 5% between days 28–34, and not rising above 40% thereafter. Treatments administered in 2004 and 2005 appeared to take longer to reach maximum efficacy, however mean abundance did fall below 17% and 30% of pre-treatment abundance respectively. In contrast to all other years, lice abundance in 2006 remained above pre-treatment levels until around 5 weeks after treatment intervention. While abundance did drop to 35% of pre-treatment levels between days 56 and 62, it remained above 40% in all other weeks.

**Figure 3:**
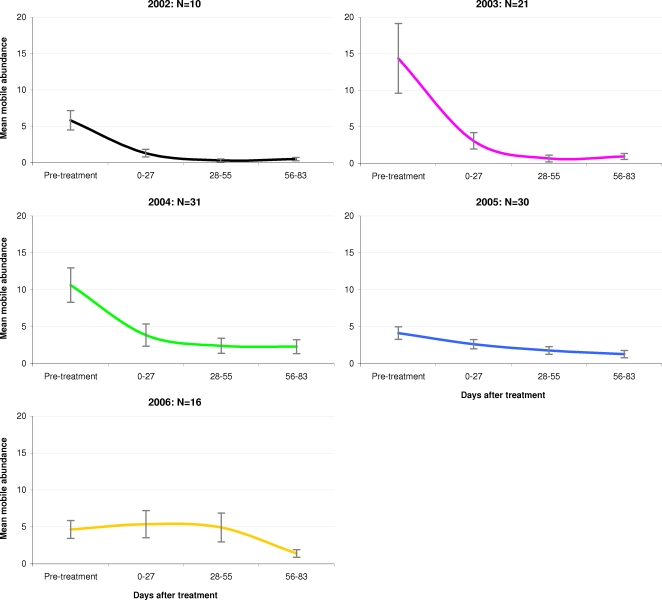
Mean abundance of mobile *Lepeophtheirus salmonis*, pre and post emamectin benzoate treatment, between 2002 and 2006. Mean mobile *L. salmonis* abundance (±SE), pre-treatment and 0–83 days after commencement of treatment. Plots based on data from 108 treatment episodes at 47 Atlantic salmon farms in Scotland in the period 2002 to 2006.

The percentage changes, as presented in Figure 2, should be considered together with the absolute mean lice abundance prior to treatment intervention. As illustrated in Figure 3, mean lice abundance prior to treatment varied considerably over the five-year period. At 14.4 and 10.7 lice per fish respectively, the mean pre-treatment abundance of L. salmonis mobiles in 2003 and 2004 was approximately two to three times higher than in 2002, 2005 and 2006. However, with the exception of 2006, mean abundance in all years dropped to below four lice per fish within a month of treatment initiation.

### Statistical modelling of post-treatment lice abundance

As was observed in Figures 2 and 3, efficacy appeared to vary amongst years. However, it was important to consider other factors that may have influenced post-treatment abundance such as geographical region, season and stage in the production cycle.

As shown in Table 3, between 2002 and 2006 emamectin benzoate treatments were administered to different ages of fish, at various times of the calendar year and across the North, South and Western Isles regions of the west coast of Scotland. Some of the treatments were loch-wide while others were for single farms only. The statistical model that was developed as part of this study (Table 4) formally investigates the effect each of these factors had on the post-treatment abundance of mobile L. salmonis lice. To take account of differing levels of lice challenge, pre-treatment lice abundance was also included as a covariate in the model.

**Table 3:** Percentages of emamectin benzoate episodes classified according to each of the variables included in the GLM.

Variable	Class	% of treatments (N = 108)
#Days after treatment	7–13	59%
	14–20	68%
	21–27	66%
	28–34	56%
	35–41	67%
	42–48	59%
	49–55	61%
	56–62	58%
	63–69	52%
	70–76	47%
	77–83	42%
Region	North	36%
	South	33%
	Western Isles	31%
Year	2002	9%
	2003	19%
	2004	29%
	2005	28%
	2006	15%
Production year	1st	45%
	2nd	55%
Season	Spring (Feb–Apr)	28%
	Summer (May–Jul)	30%
	Autumn (Aug–Oct)	30%
	Winter (Nov–Jan)	13%
Effective	No	18%
	Yes	82%
Loch-wide	No	53%
	Yes	47%

# - “% of treatments” for the variable “Days after treatment” refers to the percentage of treated farms that were monitored for sea lice in each 7-day time period following treatment.

**Table 4:** Results of the GLM analysis of sea lice abundance following emamectin benzoate treatment.

			Post-treatment mobile abundance	
Variable	***p*** **-value**	Class	Least squares mean	95% CI
# Pre-treatment mobile abundance	0.00	-	-	-
Days after treatment	0.00	7–13	3.83	[3.23–4.51]
		14–20	2.84	[2.40–3.34]
		21–27	2.29	[1.89–2.74]
		28–34	2.19	[1.80–2.65]
		35–41	2.39	[1.98–2.86]
		42–48	2.24	[1.83–2.71]
		49–55	2.24	[1.84–2.71]
		56–62	2.52	[2.07–3.04]
		63–69	2.80	[2.29–3.38]
		70–76	2.97	[2.42–3.61]
		77–83	2.94	[2.37–3.61]
Region	0.00	North	2.08	[1.79–2.39]
		South	3.10	[2.68–3.58]
		Western Isles	2.80	[2.40–3.24]
Year	0.00	2002	1.96	[1.53–2.47]
		2003	2.48	[2.05–2.97]
		2004	2.97	[2.61–3.37]
		2005	2.61	[2.28–2.98]
		2006	3.27	[2.74–3.87]
Production year	0.00	1st	2.30	[2.00–2.63]
		2nd	3.00	[2.60–3.44]
Season	0.00	Spring (Feb–Apr)	3.24	[2.52–4.10]
		Summer (May–Jul)	2.13	[1.67–2.67]
		Autumn (Aug–Oct)	2.10	[1.75–2.49]
		Winter (Nov–Jan)	3.24	[2.61–3.98]
Effective	0.00	No	7.01	[5.95–8.22]
		Yes	0.65	[0.55–0.75]
Loch-wide	0.00	No	2.16	[1.89–2.45]
		Yes	3.17	[2.79–3.59]
Region×Year	0.00	See Figure 4		
Season×Effective	0.00			
Season×Loch-wide	0.00			

Table indicates those variables found to be statistically significant (p<0.05) within the model and provides estimates of post-treatment mobile *L. salmonis* abundances and their 95% confidence intervals.

# - Forced co-variate with coefficient of 0.16 (95% CI [0.11–0.22])

In each of the seven-day time periods following treatment that were analysed, 42% to 68% of treated farms reported lice levels. Lice counts appeared to become slightly less frequent after day 62, however it should be noted that around 20% of emamectin benzoate episodes were followed by an additional sea lice treatment within the 12 week period and any lice counts recorded after such an event were discounted from the analysis.

**Figure 4:**
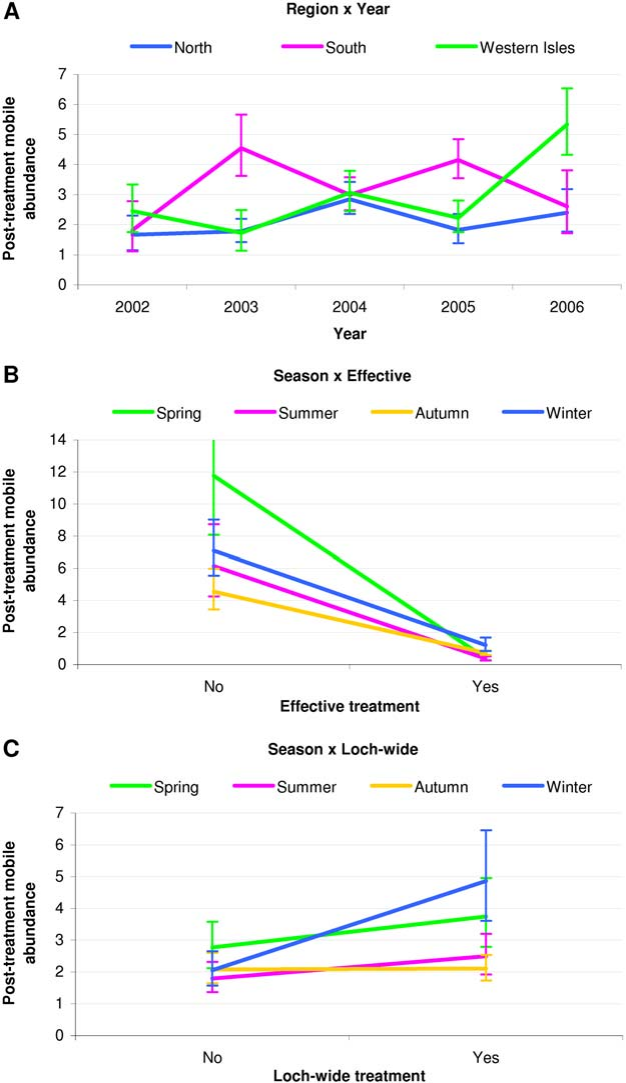
Profile plots for treatment episodes showing post-treatment mobile abundances associated with significant (p<0.05) interaction factors. Post-treatment mobile *L. salmonis* abundances with associated 95% confidence intervals. Based on a GLM analysis of data from 108 treatment episodes at 47 Atlantic salmon farms in Scotland in the period 2002 to 2006.

Table 4 gives the results of a GLM analysis of mean sea lice abundance following emamectin benzoate treatment intervention, based on all 108 episodes. All variables were found to be statistically significant (p<0.01) and are listed along with least squares estimates of post-treatment mobile abundances and their associated 95% confidence intervals. Significant interaction factors are also listed and are further illustrated in Figure 4.

The factors included in the model accounted for 66% of the variation observed amongst post-treatment sea lice abundance. Lice levels per fish subsequent to an ineffective treatment (7.01, CI [5.95–8.22]) were around 10 times higher than those observed following an effective episode (0.65, CI [0.55–0.75]) (Table 4). Overall, mean louse abundance was lowest between days 21 and 62 and levels recorded during this time were found to be significantly lower than those observed between day 7 and 13 (p<0.01). Post-treatment lice abundance was also found to be significantly higher in the second year of production when compared to the first (p<0.01).

Three significant interactions were found amongst the variables studied. Figure 4A highlights the differences that were observed between the geographical region in which the treatment was applied and the year it was administered. Post-treatment abundance in the Western Isles in 2006 (5.34, CI [4.33–6.53]) was significantly higher (p<0.05) than in any other region and year combination, except the South in 2003 (4.55, CI [3.62–5.66]) and the South in 2005 (4.15, CI [3.55–4.84]). Post-treatment abundance in the North, for all years, was significantly lower (p<0.05) than these three region and year values, except for 2006 (2.40, CI [1.77–3.18]), when it did not differ significantly from the South in 2005.

Analyses showed that ineffective spring treatments (11.77, CI [8.07–16.97]) performed significantly worse (p<0.01) than ineffective treatments applied in autumn (4.56, CI [3.44–5.97]) (Figure 4B). However, only two of the 30 spring treatments analysed were categorised as ineffective and the wide confidence interval associated with the spring value indicates that this result should be treated with caution.

A significant interaction also existed between the season in which the treatment was applied and loch-wide interventions (Figure 4C). Lice abundance following winter loch-wide treatments (4.87, CI [3.61–6.46]) was found to be significantly higher (p<0.05) than after any emamectin benzoate intervention applied in other seasons, except for loch-wide treatments applied in spring (3.75, CI [2.79–4.95]).

## Discussion

By adopting an epi-informatics approach and examining a large historical data set, the efficacy of emamectin benzoate treatments administered over a five year period, at different stages of production and across three Scottish regions was shown to vary significantly. While commercial farm records provide a rich source of information, working with production data can present some challenges. Only when a large data set is available is it possible to extract data of sufficient quality and quantity to perform meaningful analysis. The routine lice monitoring programme that was in place allowed 58% of the site-wide emamectin benzoate treatments administered between 2002 and 2006 to be included in the efficacy analysis. Two previous studies that used production data to assess the efficacy of emamectin benzoate in British Columbia [23] and on the Maine coast [28] encountered similar challenges, with fewer than 20 treatment episodes remaining (around 50% of the initially available episodes in each case) after appropriate data screening criteria had been applied. While both of these North American studies presented a snapshot of treatment efficacy within their respective geographical regions, neither attempted to show whether the efficacy of emamectin benzoate had changed over a period of time.

Prospective efficacy studies tend to be based on a relatively small numbers of treatment episodes. However, they are normally strictly controlled and often have complete and balanced data sets collected by a dedicated group of individuals working to a standard clinical efficacy protocol [20], [33]. While laboratory based studies were essential in establishing the efficacy of emamectin benzoate, they may not accurately reflect its performance under commercial conditions. A number of case studies have also been conducted to assess emamectin benzoate efficacy in the field; however several had to medicate the untreated “controls” with alternative ectoparasiticides before the planned completion date in order to avoid unacceptably high levels of infestation [21], [22], [34]. Intervening in efficacy trials in this way is sometimes necessary for the welfare of fish, but it also complicates the comparability of subsequent efficacy parameters [28]. Despite the fact that production data are rarely as complete or balanced as that collected within a prospective clinical study it can give a better indication of medicinal efficacy under production conditions and over a longer period of time, providing sufficient treatment episodes are available for analysis. While the lice counts in this study were performed by various individuals on numerous farms, it should be noted that they were conducted by qualified personnel who had been given training in this procedure. However, as pre-treatment lice counts were based on records taken from up to 16 days prior to emamectin benzoate intervention, it is likely that lice levels were sometimes higher than the baseline figures used in the efficacy calculations.

All partial site, staggered and mixed treatment episodes were screened out of this study, but it was important to further categorise the episodes that were included. Strategic sea lice treatments form part of the integrated health management programme adopted by Scottish salmon producers and not all treatment episodes are administered in response to a substantial sea lice challenge. It should also be noted that treatment-trigger guidelines are not uniform amongst countries. Despite the challenges encountered in classifying treatments as strategic or ineffective, their inclusion in this study was important as they were evident throughout the five year period. For the purposes of analysing loch-wide treatments it was important to ascertain whether other farm operators treated their respective sites for lice at the same time as the industrial partner in a coordinated manner. This information was not always available.

Alternative strategies for assessing the efficacy of emamectin benzoate were considered, but none was found to be as robust as the methodology adopted. Counting the number of treatment interventions in each production cycle or calculating the mean number of days between treatments gives an indication of treatment efficacy and lice challenge within a site. However, the former method is better suited to regions where only one sea lice medicine is available for use [23] as it is difficult to compare in-feed emamectin benzoate treatments alone with emamectin benzoate and topical cypermethrin treatments. Assessing efficacy over the entire production cycle can also become complicated if stocks are split between sites for the second year of production or when the length of time that fish are at sea varies between sites and years. While useful, these assessments are better suited to macro level analyses and may be explored in a more general risk factors study. The purpose of this study was to focus on specific issues relating to emamectin benzoate interventions in Scotland since regular treatment began in 2002.

In common with results observed in laboratory and field based trials conducted in the period 1997 to 2002 [20]–[22], [26], [27], [33], [34], the majority of emamectin benzoate treatments administered in this study significantly reduced infestations of mobile L. salmonis. However, the efficacy of treatments was not uniform between years or geographical regions. In particular the frequency with which interventions appeared to be ineffective increased toward the end of the study period.

Time to maximum efficacy varied widely across episodes, but generally it was reached 28–34 days post treatment initiation and overall levels of lice were lowest between days 21 and 62. This is broadly in line with findings from a recent study on the Maine coast by Gustafson and colleagues that also used pre-treatment lice loads to calculate the efficacy of emamectin benzoate [28]. However, it should be noted that of the 19 treatments analysed in the Gustafson study, all were found to be more than 60% efficacious and pre-treatment abundance was generally lower, particularly when compared to the levels observed in Scotland in 2003 and 2004.

Duration of efficacy has been reported to be as long as five months in the Broughton Archipelago region of British Columbia [23] although some lice re-infection occurred after three months. In the current analysis, post-treatment lice levels in Scotland generally remained below pre-treatment abundance for the full 83 days examined, but began to rise around week nine. It is interesting to note that, on average, the levels of lice observed prior to intervention were similar in both studies, but that farms in British Columbia were re-treated less often than those in Scotland. However, it is likely that the large populations of migratory Pacific salmon found in the waters of British Columbia create very different dynamics in terms of sea lice challenge than those in Scotland.

The differences found between the present study and that conducted in Scotland in 2002 [27] are of particular interest. In the earlier study [27], zero levels of lice were attained for 12–14 weeks post-treatment, however it should be noted that this small scale trial involved only two farms and three emamectin benzoate treatment episodes. Nevertheless, the results presented herein may indicate that emamectin benzoate is not as effective on Scottish salmon farms as it once was. Direct comparisons with other studies are less straightforward, either because efficacy was monitored over a shorter period of time [22], [26], [33] or because untreated cohorts provided a potential source of re-infestation, that may have extended the time taken to reach maximum efficacy or reduced the duration of efficacy [21], [34].

Previous analysis has shown that since 2002 L. salmonis mobile abundance tends to vary between geographic regions in Scotland [19], with farms in the North generally experiencing lower levels of infestation. The present study shows that post-treatment lice abundance also differs between regions. Again, levels in the North were found to be lower and increases in post-treatment abundance in this region in 2004, and in the Western Isles in 2006, match similar trends observed in overall mobile lice levels [19]. The peaks and troughs observed in post-treatment abundance in the South did not closely match patterns of mobile infestation in this region, confirming that variables other than year and region are important and should be considered in a fuller analysis of all risk factors.

It is known that seasonal variation occurs in lice infestation on Scottish farms and that abundance is generally higher in the second year of production [13], [14], [19]. It is perhaps then unsurprising that post-treatment lice levels also varied throughout the production cycle in the present study. The univariable analyses carried out by Gustafson et al [28] found that treatments administered to larger fish required more time to reach maximum efficacy. In this multivariable study no significant interaction was found between production stage and days after treatment, but post-treatment abundance levels were higher in second year fish. While the relative performance of emamectin benzoate treatments across the seasons varied, it appears that post-treatment levels were highest following winter (Nov–Jan) and spring (Feb–Apr) treatments. Given that all but one of the winter treatments occurred in the first production year and nearly all spring treatments occurred in the second, it appears that treatments applied in the middle six months of the production cycle may not be as efficacious as those administered during other periods. However, it should be noted that the distribution of treatments throughout the production cycle was not balanced. Furthermore, significant interactions were found between season and effective treatments as well as between season and loch-wide treatments, making it difficult to conclude what effect season has on treatment efficacy.

Reduced sensitivity, and potential resistance, of sea lice to currently available medicines remains an important area for continued research. Further analysis regarding the structure of lice populations prior to and following treatment needs to be undertaken to assess whether it has any bearing on efficacy. The prevalence of sea lice following treatment intervention should also be examined to ascertain whether lice that persist post-treatment are present on many or few fish within a farm. Studies that compare alternative treatment strategies across the whole production cycle are also important [27] as product rotation will prevent over-dependence on one sea lice medicine and may discourage resistance in the long term.

The ineffective treatments reported in this study may have lacked efficacy for a number of reasons relating to fish appetite and feeding rate, sub-therapeutic dosing due to underestimated biomass or variation in medicine inclusion level in the feed, amongst others. *In vivo* methods for assessing efficacy exist, but the techniques used for the bioassay of emamectin benzoate require further validation. Furthermore, the viability of lice post-treatment is often compromised and therefore improved methods for enumerating attached individuals, including characterisations such as “moribund lice” or “non-viable egg-strings”, must be developed [35]. These concerns have led to the establishment of a scientific group to advise the Scottish salmon industry on a protocol for, “monitoring sea lice for resistance to approved treatments” (Scottish Salmon Producers Organisation, Minutes of the Integrated Sea Lice Management group, 30^th^ August, 2007; Perth, Scotland). In summary, all concerned parties must continue to closely monitor treatment interventions before a definitive assessment can be made as to whether the efficacy of emamectin benzoate against infestations of sea lice on Atlantic salmon farms in Scotland is diminishing.
